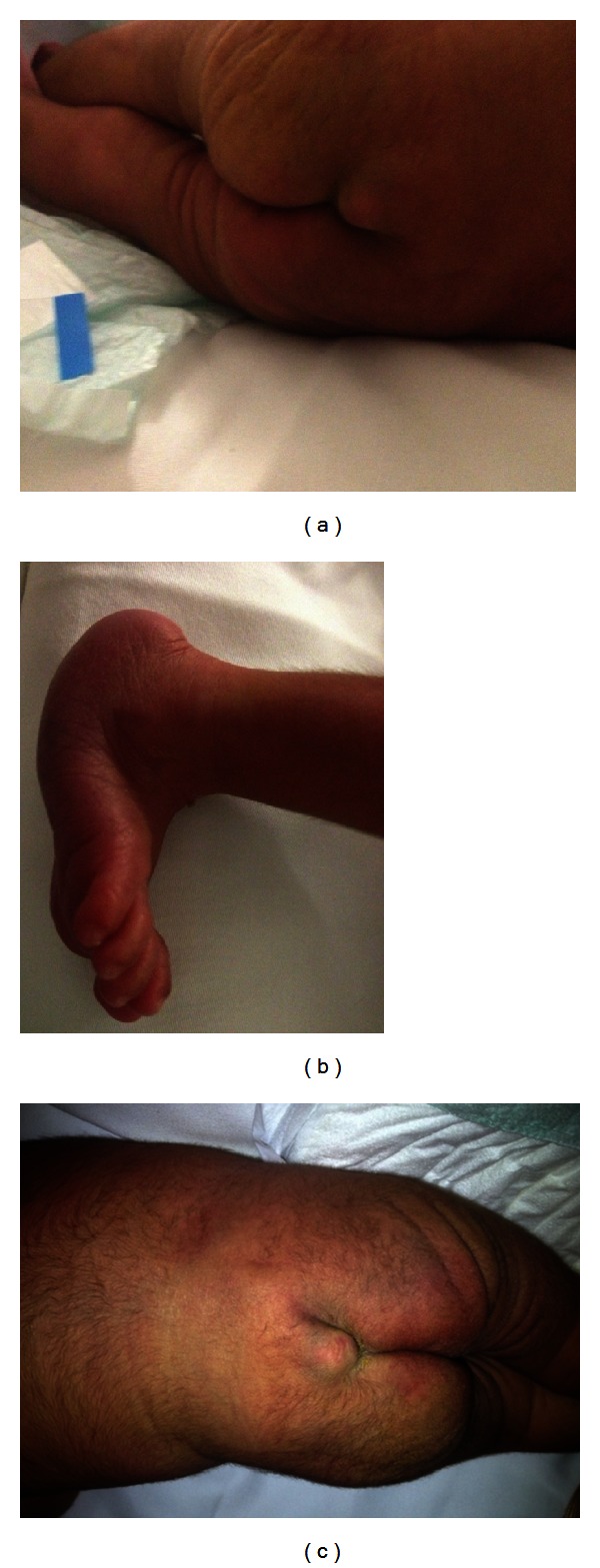# A Sacral Mass in a Newborn: A Variant of Type II Diastematomyelia with Triple Splitting of the Cord

**DOI:** 10.1155/2013/307205

**Published:** 2013-05-08

**Authors:** Emad Sadek Shatla, El Said M. A. Bedair, Ashraf Soliman

**Affiliations:** ^1^NICU, Hamad Medical Center, P.O. Box 3050, Doha, Qatar; ^2^Radiology Department, Hamad Medical Center, P.O. Box 3050, Doha, Qatar; ^3^Pediatrics Department, Hamad Medical Center, P.O. Box 3050, Doha, Qatar

## Abstract

Ultrasonographic (US) evaluation of spinal anomalies is limited. MRI is increasingly being used in the diagnosis of spinal anomalies. MRI has been able to show clearly the detailed anatomy of this rare case of type II diastematomyelia with triple splitting of the cord.

## 1. Introduction

Diastematomyelia, also known as split cord malformation (SCM), is a congenital spinal anomaly in which there is longitudinal splitting of the spinal cord. Females are affected much more commonly than males [1]. This condition occurs in the presence of an osseous (bone), cartilaginous, or fibrous septum in the central portion of the spinal canal which then produces a complete or incomplete sagittal division of the spinal cord into two hemi cords. When the split does not reunite distal to the spur, the condition is referred to as a diplomyelia or true duplication of the spinal cord.

MRI is a safe and important tool for confirming the presence of spinal cord abnormalities, when a suspected spinal deformity is detected on US. MRI clearly demonstrated the lesion in this rare case after suspecting spinal abnormality (diastematomyelia) by US [2–10].

## 2. Case Report

37-week we have term female delivered by LSCS. APGAR score was 9 and 10 at 1 and 5 min, weight was 1890 grams, length was 43 cm, and head circumference was 31 cm. At birth baby needed no active resuscitation. Ten mins after delivery she developed an episode of apnea which required bagging for 2 min. Baby was shifted to NICU, connected to nasal CPAP for few hours, and then settled. On examination she had a soft midline sacral swelling in the sacral area (Figures 6(a) and 6(b)) with rocker bottom feet (Figure 6(b)), low set ear, and a small PFO with left to right shift diagnosed by echocardiography. Chest X-ray showed abnormal ribs. ultrasonography of the brain and abdomen was normal. Ultrasound spine showed double-spinal cord (diastematomyelia). MRI was done on day 4 of the neonatal period and showed the rare deformity of triple splitting of the cord. 

**Figure 1:**
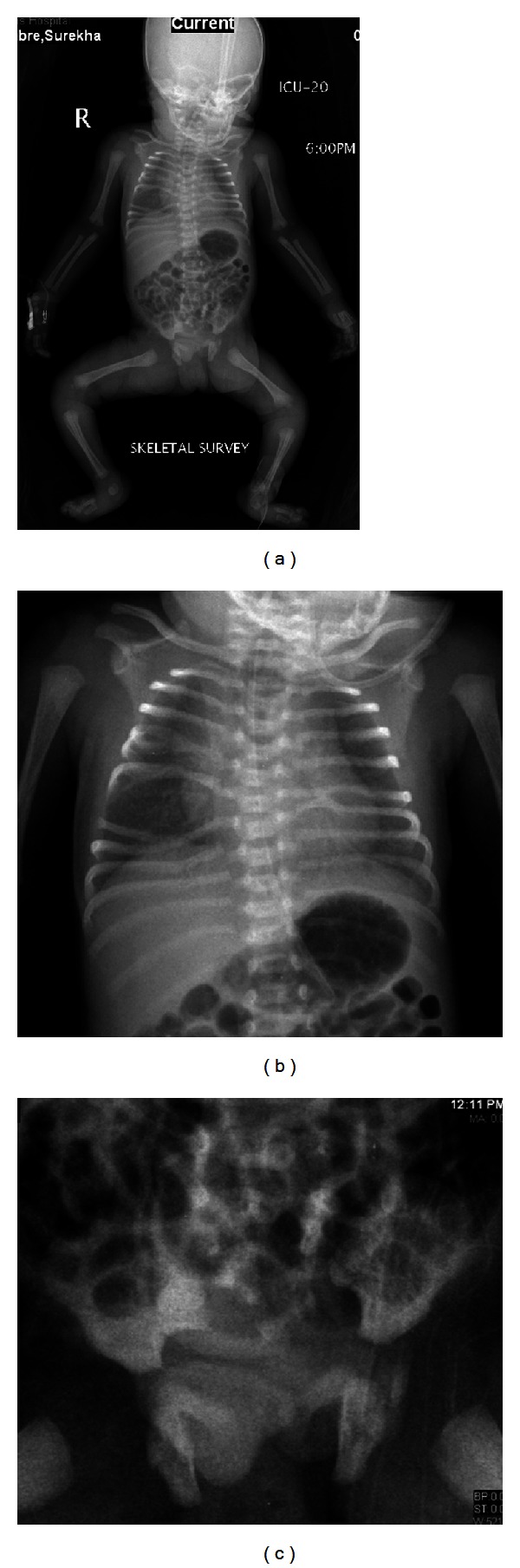
Babygram (a) and magnified chest area (b) and pelvic area (c); there is abnormal orientation of the ribs with splitting of right 5th and 6th ribs and absent posterior part of right 7th rib with marked splaying and spacing between 6th, 7th, and 8th ribs. Partial agenesis of the sacrum (lower segments) more involving the left side of the sacrum with associated deformity of adjacent left iliac bone and SI articulation, widening of interpedicular distance of lower dorsal and lumbar spines; no other skeletal abnormality identified.

**Figure 2:**
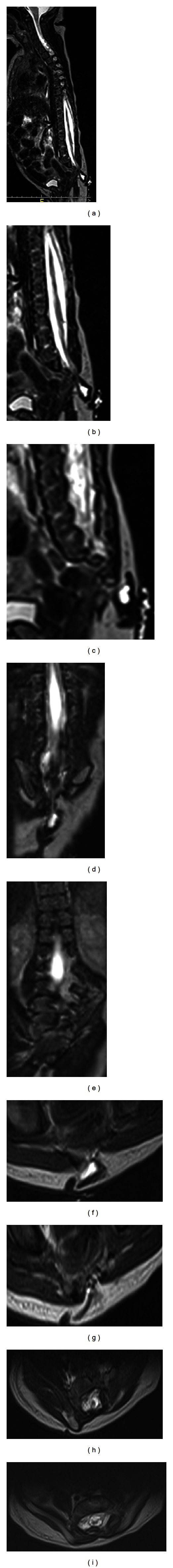
MRI T2 sagittal (a, b, c), axial (f, g, h, i), and coronal (d and e), for the spine demonstrating widening of bony spinal canal and 2ry dural ectasia (short arrows), abnormal sacral segmentation (bold arrows) and partial sacral agenesis and hypoplasia (long arrows), deformity of the iliac bone (circle), the meningocele (arrow head), and the fibrous band (curved arrow) related to it.

**Figure 3:**
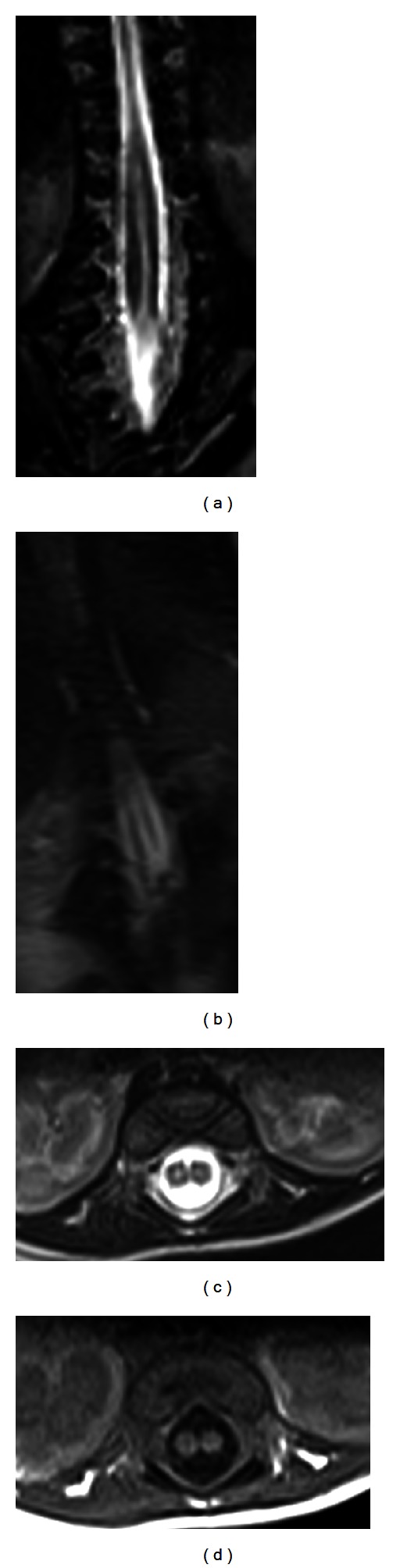
T2 coronal (a and b), T2 axial (c), and T1 axial (d) for spines demonstrating the spitted cord (diastematomyelia).

**Figure 4:**
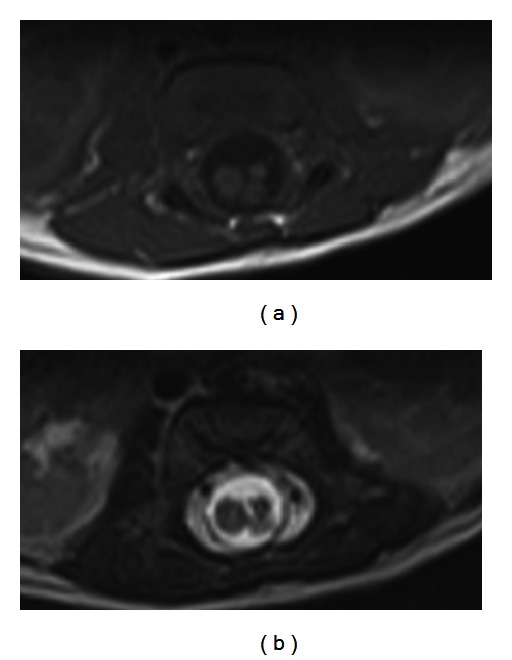
Axial T1 (a) and T2 (b) of lower lumbar region demonstrating the triplet splitting of the cord noted that the left cord is the one resplitted.

**Figure 5:**
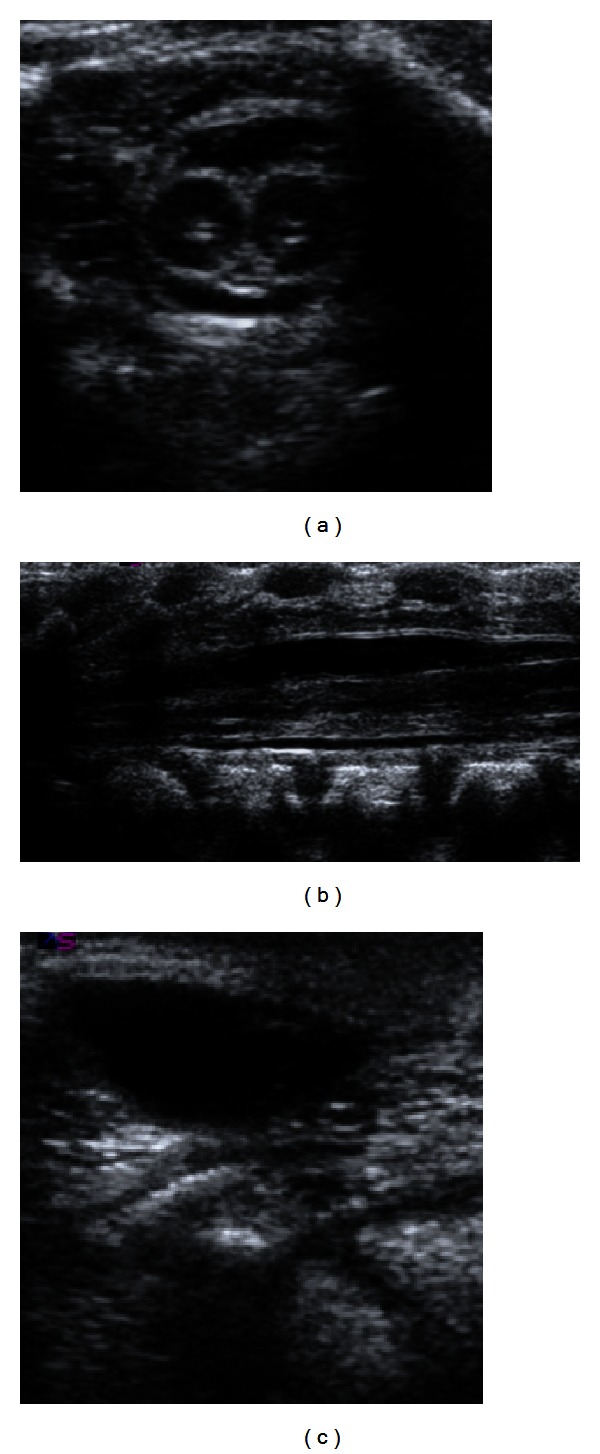
US for the spines: (a) TS scan for cord at upper lumbar region demonstrating the splitted cord (diastematomyelia), (b) LS scan at lumbar region showing the dural ectasia and the cord splitting in long axis, (c) demonstrating the meningocele at lower sacral region.

Plain skeletal survey (baby gram), US brain, abdomen, spine, and hips, and MRI brain and spines were done for the newborn and demonstrated the followings: (1) partial asymmetrical sacral agenesis with slightly deformed left sacroiliac articulation and partial hypoplasia of the left iliac bone corresponding to the hypoplastic articulating sacral segments. Only the 1st sacral segment is well developed, while the 2nd, 3rd, and 4th segments were poorly developed specially the left aspect, and the 5th segment was absent, this was associated with partially opened posterior sacral canal (Figures 1 and 2). (2) A sizable meningocele was attached to the distal sacral canal with relatively thick fibrous band passing through the meningocele to skin at right side of midline, and cranially it was attached to the right filum terminale (Figure 2). (3) There were tethering and splitting of the cord (diastematomyelia) extending from the lower dorsal level to the conus medullaris opposite LV4, and the splitting involved the filum terminale. There was no bony or cartilaginous septum identified (Figure 3). There were another 2nd splitting of the left-sided cord near its distal end to form a triplet cord appearance for short segment; then it is rejoined again just above the left filum terminale (Figure 4). In addition evident dural ectasia was noted with very capacious lumbar canal extending downwards from the lower dorsal to sacral segments (Figure 2). There was segmentation abnormalities in the form of deformed orientation and development of some ribs (few were splitted and others have absent posterior segments) but still 12 ribs were identified (Figure 1). US examination was successful to depict the presence of the splitted cord and meningocele (Figure 5).

## 3. Discussion

 The signs and symptoms of diastematomyelia may appear at any time of life, although the diagnosis is usually made early. Cutaneous lesions (or stigmata), such as a hairy patch, dimple, hemangioma, subcutaneous mass, lipoma, or teratoma, override the affected area of the spine which is found in more than half of the cases. Neurological symptoms are nonspecific, indistinguishable from other causes of cord tethering. The symptoms are caused by tissue attachments that limit the movement of the spinal cord within the spinal column. These attachments cause an abnormal stretching of the spinal cord [3, 4].

Diastematomyelia may be an isolated finding or may be associated with other spinal dysraphisms such as myelomeningocele, meningocele, lipoma, neurenteric cyst, and dermal sinus. The vertebral anomalies associated with diastematomyelia include hemivertebra, with kyphosis or scoliosis. There may be associated renal, rectal, and uterine malformations. The most common location of diastematomyelia is in the thoracolumbar region. Rarely, it can affect the cervicodorsal region. 

In our patient, the dorsolumbar diastematomyelia was type II. However, because of lack of antenatal care, this was not prenatally detected. There was no associated dermal sinus in this patient. MRI showed the lesions better and with less interobserver variation than USG as in all patients with suspected spinal anomalies. If US diagnoses the lesion antenatally fetal MRI should be used prior to further management [5–7]. 

In summary, triple splitting of the cord is very rare anomaly that can be diagnosed accurately using clinical, ultrasonographic, and MRI evaluations [9]. 

## Figures and Tables

**Figure 6:**